# Digital pathology structure and deployment in Veneto: a proof-of-concept study

**DOI:** 10.1007/s00428-024-03823-7

**Published:** 2024-05-14

**Authors:** Albino Eccher, Stefano Marletta, Marta Sbaraglia, Angela Guerriero, Mattia Rossi, Giovanni Gambaro, Aldo Scarpa, Angelo Paolo Dei Tos

**Affiliations:** 1 Department of Medical and Sciences for Children and Adults, University of Modena and Reggio Emilia, University Hospital of Modena, Modena, Italy; 2 Department of Diagnostic and Public Health, Section of Pathology, University of Verona, P.Leee L.A. Scuro N. 10, 37134 Verona, Italy; 3 Division of Pathology, Humanitas Istituto Clinico Catanese, Catania, Italy; 4 Surgical Pathology and Cytopathology Unit, Department of Medicine-DIMED, University of Padua School of Medicine, Padua, Italy; 5 Division of Nephrology, Department of Medicine, University of Verona, Verona, Italy

**Keywords:** Digital pathology, Automatization, Standardization, Whole slide imaging, Artificial intelligence, Healthcare networks

## Abstract

Nowadays pathology laboratories are worldwide facing a digital revolution, with an increasing number of institutions adopting digital pathology (DP) and whole slide imaging solutions. Despite indeed providing novel and helpful advantages, embracing a whole DP workflow is still challenging, especially for wide healthcare networks. The Azienda Zero of the Veneto Italian region has begun a process of a fully digital transformation of an integrated network of 12 hospitals producing nearly 3 million slides per year. In the present article, we describe the planning stages and the operative phases needed to support such a disruptive transition, along with the initial preliminary results emerging from the project. The ultimate goal of the DP program in the Veneto Italian region is to improve patients’ clinical care through a safe and standardized process, encompassing a total digital management of pathology samples, easy file sharing with experienced colleagues, and automatic support by artificial intelligence tools.

## Introduction

In the era of targeted medicine, pathologists must deal with an increasing daily workload to assess extremely detailed diagnoses to guide specific patient-designed therapies. In this scenario, digital pathology (DP) and whole-slide imaging (WSI) represent disruptive technologies with great potential to meet the needs of the modern medical community. These technologies not only allow pathologists to quickly share challenging cases with experienced, remotely located colleagues to get second opinions but they also enable scanned slides to be stored in virtual archives from where they may be easily and quickly retrieved [1]. Furthermore, DP and WSI perfectly suit the application of artificial intelligence (AI)-based algorithms, which can improve the accuracy of diagnosis and the development of personalized treatment plans, assisting clinicians with decision-making. Also, AI can provide healthcare systems across multiple locations with the fundamental process of slide screening to recognize morphological patterns to quickly guide proper diagnosis and therapy [2]; however, despite great strides in recent years, the adoption of DP remains limited to only a few pathology laboratories. While generally exploited for research purposes [3, 4], DP has also been progressively employed for primary diagnoses by several institutions [5, 6]. Such innovative tools have been variably adopted either by pathology laboratories alone [7] or by complex healthcare networks of hospitals [8], like as in South Tyrol, Italy [9], and the DigiPatICS project in Catalonia, Spain [10]. These networks, however, have found that connecting an extensive network of hospitals is a huge challenge. Having a group of modern but different hospitals sharing a unique model may be complex in terms of identifying proper hardware (scanners and displays etc.) and software tools for each laboratory. Furthermore, despite technological progress increasing the application of DP and its affordability, the pathology community’s widespread adoption of this technology for primary diagnosis is still lagging behind [11], hampered by technical and managerial issues [12]. These considerations are of particular concern for specific fields, such as cytopathology, which still lack broadly acknowledged validating guidelines [13].

**Fig. 1:**
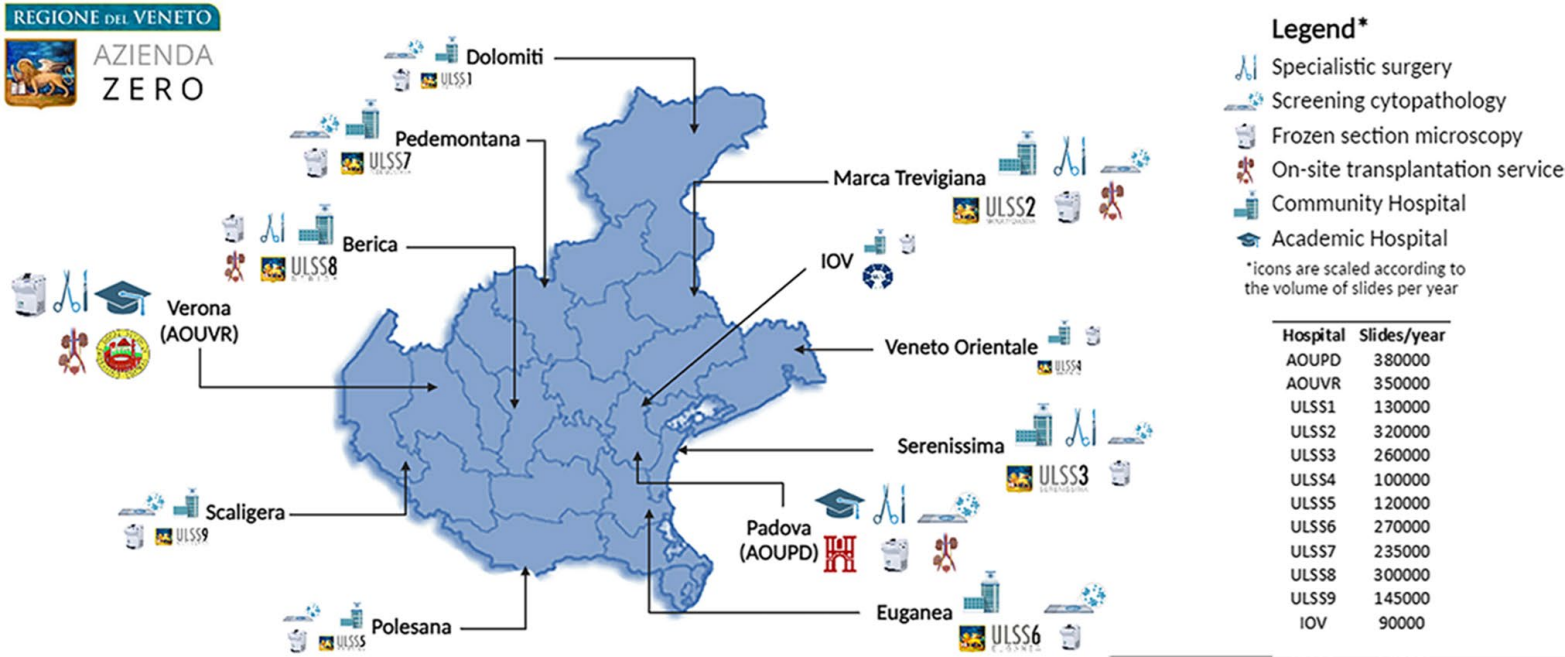
Map of the Veneto region displaying the location of the twelve involved hospitals and the services they provide

Azienda Zero is the broadest public healthcare provider in Veneto’s North Italian region and almost 5 million people benefit from the Azienda Zero healthcare system. It is organized into two leading academic hospitals (The University of Verona and Padova), a comprehensive cancer center/research (“Istituto di Ricovero e Cura a Carattere Scientifico”) hospital (“Istituto Oncologico Veneto;” IOV), and nine hospital networks (“Unità Locale Socio Sanitaria;” ULSSs); these latter networks include 26 smaller local districts. As far as pathological anatomy is concerned, the nine ULSSs, the two academic hospitals, and the IOV collect samples from institutions located throughout the entire Veneto territory [14]. While all of them are equipped with basic facilities, such as intraoperative frozen-section microscopy, a few hospitals provide more specific services. For example, (i) pathological samples from specialist surgery are analyzed by the laboratories of the two academic hospitals and three community hospitals; (ii) the Padova University Hospital and seven ULSSs gather cytological material from the whole region for the cervical cancer screening program, and (iii) an on-call pathologist is available 24/7 in the two academic hospitals and two community hospitals for intraoperative evaluation of transplantation specimens. The routine workflow of such laboratories globally accounts for about 240,000 slides per month and nearly 3 million slides per year (Fig. 1). In light of the above, the pathology laboratories in the Veneto region suit the adoption of a widely shared DP system. The aim of the present work is to describe the planning and effort required to enable the network of pathology laboratories in Veneto to go fully digital. In the following sections, we detail the characteristics of the hardware and software components considered and related to clinical needs, describing this crucial step of the digital transformation journey for pathological anatomy, providing a structured approach to assess feasibility, ensure compliance, and optimize the implementation of DP in the healthcare domain.

## Context and methods

The DP program was supported with European funds to optimize pathological diagnosis in the network of public hospitals in the Veneto region. After a comprehensive assessment of data and needs provided by individual hospitals, the regional governance outlined a strategic framework with specific clinical needs. A market investigation called “Technologies for Digital Pathology” was carried out to verify the presence of appropriate tools tailored to the network’s needs. We want to stress that although technological tools, such as scanners and displays, represent one of the main points of the “digital pathway,” going fully digital is not only about buying tools; the transformation of a conventional pathology laboratory should encompass all the processes and the people routinely working there, who must be adequately trained to embrace the cultural change. Considering this step as mandatory for each laboratory, in the following sections, we describe the characteristics of the items necessary to realize the project according to the clinical needs.

### Image-acquisition system

Scanners are essential for the digital workflow, and a variety of models are now available on the market [15]. Consultations with various suppliers were planned according to the predicted services for each community and academic hospital. Four scanner categories were selected based on their specific technical characteristics:

- Batch 1: high-throughput scanners. Such devices were meant to satisfy the needs for routine diagnosis in high-volume institutions (i.e., the academic hospitals of Verona and Padova, and the community hospitals of Treviso, Venezia, and Vicenza) provided by high-volume specialistic surgical services. Therefore, these had to be capable of quickly and accurately digitizing a large number of hematoxylin and eosin (H&E)-stained slides and immunohistochemistry (IHC) preparations.
- Batch 2: medium-throughput scanners. For the smaller community hospitals, there would have been no point in acquiring expensive hardware, performing far beyond the required daily workflow. Instead, these hospitals were more likely to benefit from less-expensive medium-capacity scanners, proficiently supplying the demands of their inpatient and outpatient services.
- Batch 3: low-throughput scanners. This type of scanner was intended to address the two critical tasks of pathology laboratories: fluorescent microscopy and on-site intraoperative examination, both required for transplantation surgery. Such services would not have benefited from the bulky and heavy scanners commonly employed for routine diagnosis, as they usually deal with only a small number of slides. Conversely, light (but still fast) devices better suit these applications. For transplantation, quick digitization also allows physicians to share challenging cases with remotely located colleagues, which can help them to address the pressing demands of the operating theater.
- Batch 4: cytopathology. Digitalization of cytological material always carries some issues linked to the peculiar characteristics of these specimens. Compared to histology, cytological material does not often homogeneously distribute onto slides, so scanning of multiple focal planes (“Z-stacking”) is often required to obtain appropriate WSIs [16]. The Z-stacking process typically results in longer scanning times and larger digital files. To overcome this problem, some companies have incorporated enhanced methods of Z-stacking into their products, for example, software selecting and combining the sharpest image from each focus into one single image (“Extended Focus”). As screening cytology accounts for a noteworthy percentage of the daily workflow of different hospitals, specific consultations were carried out to identify the most suitable devices, potentially integrating with already available AI-based support systems [17].

### Workstation and viewing software

Digital displays (i.e., monitors) are an indispensable component of the workflow, and choosing the best display is just as important as selecting the most appropriate microscopy setup for a pathologist providing formal diagnoses. Displays vary greatly in parameters such as size, shape, esthetic design, resolution, brightness, contrast ratio, refresh rate, screen reflection, and viewing angle. A total of 230 workstations were used to let pathologists, residents, students, other professionals, and meeting attendants visualize and navigate WSIs. Each workstation was made up of a personal computer provided with a CPU i7 processor, 16 Gb RAM, 512 Gb SSD, and a full HD graphic card connected to two diagnostic medical-grade US Food and Drug Administration-approved full HD monitors (resolution 1920 × 1080 pixels), at least 27 inches in size. Users could use one display to show histological images and the other to view data. Regardless of the use case, there is a paucity of data on display specifications, particularly their definitions, how they apply to different display categories, and how the specifications can be used to help choose the right display for the intended use. Based on the tasks that they were intended for [18], we identified two categories of displays, distilled from manufacturer and computing hardware websites: (i) medical grade (MG) and (ii) consumer off-the-shelf (COTS). MG displays are typically expensive, built for multiyear use, and have standardized features to provide a uniform experience for their users. These are contrasted with COTS displays, which are general‐purpose displays. Most pathologists today use COTS displays as their primary display, provided to them as part of a standard core workstation configuration. Once scanned, pathologists can analyze displayed WSIs thanks to specific viewing software provided by the supplier, coupled with an internal laboratory integrative system (LIS). Apart from visualizing virtual slides, the viewing software allows physicians to navigate WSIs with a range of annotation functions, including drawing regions of interest, zooming in and out, rotating, and measuring.

### Laboratory integrative system (Fig. 2)

**Fig. 2:**
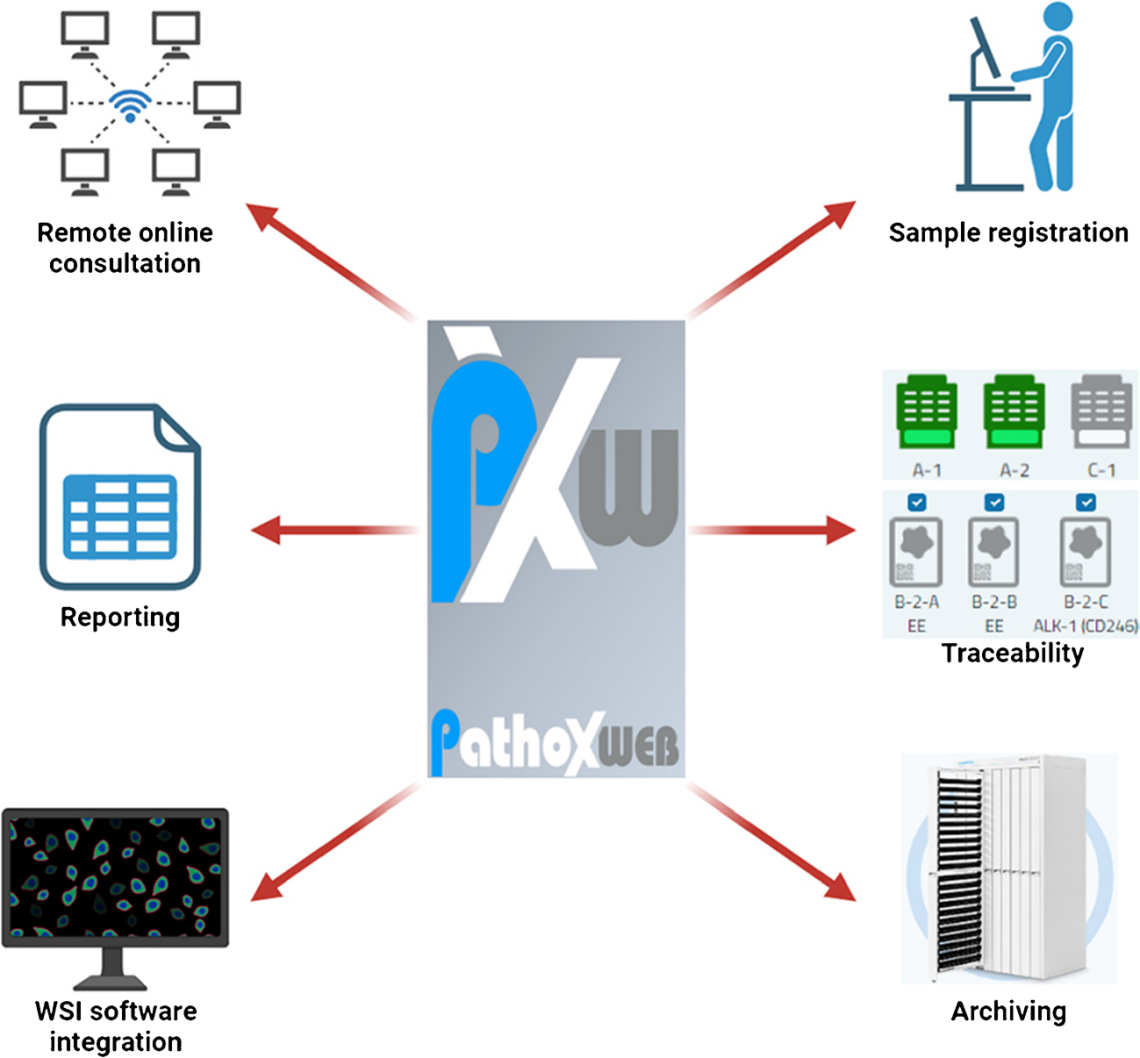
Functionalities provided by the PATHOXWEB 2.0 laboratory integrative system

One of the key points for the realization of the project was the unification of the workflow for all laboratories, which were thought of as a coordinated network. The distribution of pathology laboratories across multiple locations, with two high-volume academic hospitals, greatly suits this project. Therefore, to achieve this goal and following a market consultation, the PATHOXWEB 2.0 software provided by TESI Group was chosen as the regional LIS to be uniformly shared by all the institutions. PATHOXWEB 2.0 is a state-of-the-art updated web-based software developed to support professionals in handling the whole workflow of pathology laboratories going fully digital, from sample registration to definitive diagnosis and archiving. The web-based configuration of the system allows its remote usage on workstations and computers by any physician, technician, biologist, or other professional. Thanks to a middleware program, the software can be easily interfaced with local healthcare informatics systems and, as for the interests of a DP laboratory, has the following functions [19]:

- Acceptance of requests, check-in, and registration of samples collected from inpatient and outpatient services by reading unique barcodes and assignment of a unique identifier for the accepted exam;
- Assistance in specimen traceability and workflow quality checks in all laboratory phases. The software can be interfaced to laboratory devices that carry out sample tracking, including cassette printers, scanners, tissue processors, stainers, and gross-picture acquisition programs;
- Residual material, blocks, and slide storage and retrieval management thanks to the interaction with specifically configured archiving desks;
- Interface with WSI viewing software for supporting the visualization of scanned digital slides;
- Assisted reporting with archiving of standard sentences and use of International Collaboration on Cancer Reporting-based checklists;
- TelePathox program, a web-based consultation service allowing online correspondence of challenging cases with remotely located, experienced colleagues to get trusted second opinions.

### Storage

According to the guidelines, “tissue blocks and glass slides should be kept long enough to ensure that the patient is treated properly”. Such a period has been operatively quantified by the UK’s Royal College of Pathologists as 10 years for histology slides and smears, and the patient’s entire life for blocks [20]. Planning a storage solution for digital files requires the consideration of several factors, including the number and size of the files, the number of users who will access the files, the interface between users, the level of data protection and security, and the budget. In the context of the realization of the current project, the digital storage process of WSI focused on the following points:

- Cloud-based archiving of digital images and data in central/federated vendor-neutral archives, allowing easy and fast case identification and retrieval, planning for a long-term central archive after initial local management at the laboratory (short-term local archive). The purpose of this distinction is to streamline the management of the digital archive through centralization. It could be possible to automatically recover the slides of each patient every time there is an examination or letting the local pathologist decide which cases to keep in the short-term archive;
- Association of digitally saved data with other healthcare-related information uploaded from the internal LIS;
- Image backup option, able to provide disaster recovery and continuity exchange with external health organizations;
- Indexing research tool employing the topography and morphology Systematized Nomenclature of Medicine Clinical Terms (SNOMED CT) vocabulary.

### Artificial intelligence

The academic community of the Veneto region has always welcomed the adoption of new technologies as long as validated safety requirements support them. In pathology, as in many other scientific fields, AI is revolutionizing the approach to historically problematic issues, speeding up the decision-making process, and increasing the standardization of reports. Thus, with the goal of both enhancing diagnostic accuracy as well as reducing subjective interpretations and turn-around times, the present project chose to fully embrace the currently approved AI-based deep-learning tools, including:

- Support for prostate biopsy screening for cancer and Gleason score grading [21];
- Automatic evaluation of breast cancer IHC biomarkers (estrogen receptors, progesterone receptors, and Ki-67 proliferative index) [22];
- Computer-assisted PD-L1 scoring in lung cancer specimens [23].

The use of the abovementioned products for in vitro diagnostic purposes has been endorsed by international regulatory entities after strict evaluation of their evidence-supported safety standards and performances.

### Specialist networks

Specialist networks act to exchange knowledge, experiences and best practices, and training. In Veneto, the nephropathology network consists of numerous points of care that, however, cannot have the necessary expertise due to their diagnostic complexity. The project plans to structure reporting workstations within the network’s nephrology departments, similar to those in the pathology laboratories. This will allow the entire regional nephrology network to be connected, to manage the most complex cases, carry out process quality controls, standardize reporting, and consequently improving the therapeutic management of patients.

### System tracking, quality checks, and archiving of tissue specimens

Standardized tracking, archiving, and conservation of materials are pivotal to patient care and may significantly influence diagnostic accuracy and, ultimately, clinical management. To ensure precision and repeatability of the whole workflow, pathology laboratories involved in the project had several quality control checkpoints. Barcode-scanning equipment integrated with a local LIS was employed to provide the lowest rate possible of human-eye-caused manual mistakes. First, each specimen received by the laboratory was accompanied by an e-request providing relevant clinical data created in local electronic healthcare record systems by the sending clinicians. After registration, the sample was given a unique identification number labeled with a DataMatrix-type barcode. Such an identification code is essential to track the specimen through its entire “life” within the laboratory, notifying professionals about its status, and eventual actions required from the registration to the final released diagnosis. Specifically, (i) labels were applied to the containers for sampling and material storing; (ii) barcodes were printed on each paraffin block cassette; and (iii) labels including the DataMatrix code were automatically printed on each H&E, IHC, and/or cytological slide related to the specimen. Most hardware and computers (i.e., tissue processors, scanners and stainers, etc.) of the involved institutions will be equipped with barcode readers integrated with the LIS, enabling the working team to check the samples safely during the subsequent processing steps.

As previously mentioned, for some regulatory entities [20], the recommendations advise prolonged tissue conservation after the final diagnosis, covering the whole patient’s life. The LIS automatically records a complete audit trail of each block and slide. The system relies on a modular system, progressively adding more units to its capacity without limiting the number of collectible items for short and long storage periods.

### Validation

Validation is defined as a process that demonstrates that WSIs will perform as expected for their intended use and environment before using them for patient care. Regarding the validation of the entire digital network (scanners, displays and AI tools, etc.) for primary diagnosis, the recommendation for each institution is to follow the College of American Pathologists (CAP) guidelines [24], to ensure the safety and robustness of the project. The validation process must include a sample set of at least 60 cases for one application, or use case, that reflects the spectrum and complexity of specimen types. Several pathologists per center are experienced in DP and will add value to the validation process, randomly evaluating the selected WSIs previously assessed by conventional light microscopy (LM) after the washout period. Although all discordances between WSI and glass-slide diagnoses discovered during validation need to be reconciled, a 95% diagnostic concordance threshold for the same observer between LM and WSI will be the goal, according to the CAP guidelines. As far as cytopathology is concerned, widely acknowledged validating guidelines are still lacking [13]. Hence, an expert-based consensus on the non-inferiority of virtual to glass slides reviewed by dedicated cytopathologists was chosen as a reliable criterion for safely adopting the WSI technique in this field.

## Discussion

In the past few decades, DP and AI have represented disruptive technologies in physicians’ ways of viewing their daily work, meeting the pressing scientific community’s demand for refined and reproducible diagnoses in the modern era of precision medicine. Despite great technological progress, the adoption of DP for routine clinical diagnostic use remains limited, and the complete transition to DP of a pathology network constitutes an organizational, functional, and technical challenge. Generally, the need for significant investment, along with pathologists’ skepticism of the adoption of new technologies has hampered comprehensive DP employment by many institutions. These considerations notwithstanding, plenty of solutions supporting the complete automatization of a pathology laboratory workflow are now available [25] and the number of departments and networks fully embracing DP for primary diagnosis keeps on increasing worldwide [26]. Since the first pioneer institutions such as the Memorial Sloan Kettering Cancer Center in the USA [27], many laboratories have followed in their footsteps, resulting in the modern broad comprehensive regional systems such as the Catalonian DigiPatICS [10], among others.

Regarding Italy, WSI-based routine diagnosis has been limited to a few isolated (but still illuminating) models, such as the frozen-section telepathology service in South Tyrol [9] and the locally connected network in Eastern Sicily [7, 28]. Thus, the digital transformation of the network of public pathology laboratories in the Italian Veneto region reported herein certainly represents a milestone for the further widespread adoption of this revolutionary technology in Italy. The realization of the present project involved a network of 12 hospitals producing more than 2.5 million slides per year, with a number that is likely to increase. Although such a process has required a large amount of public funds and resources, it will remarkably decrease operational and asset costs in the coming years. Several studies have outlined an estimated saving of more than $250,000 per institution per year after the implementation of DP [15], making the achievement of such a goal in the Veneto region likely as well.

Alongside the economic aspects, the broad deployment of DP, WSI, and AI in laboratories in Veneto will play a crucial role in facilitating the exchange of information among the involved centers. Not only will this enhance the standardization and reproducibility of pathology reports but it will also help physicians establish experienced second opinions to improve overall patients’ clinical management. Apart from the evaluation of routine biopsy and surgical specimens, it is worth mentioning the implications of knowledge and opinion-sharing in two particular fields of pathology: intraoperative consultation and cytopathology. For the former, on-site evaluation of frozen sections is supposed to quickly provide surgeons in the operating theater with the most appropriate pathological response possible. Also, especially in the transplantation setting, rendering widely reproducible diagnoses is not usually straightforward due to subtle histological changes influencing diagnostics classifications. Modern DP-based cockpits significantly help overcome such an issue [29, 30], offering the opportunity to get real-time opinions from remote, experienced colleagues. Hence, the broad network of online connected hospitals will let pathologists, particularly the youngest ones, exploit a teleconsultation service to share the most challenging cases regardless of their physical location. Together with obvious advantages in terms of clinical care, this system will provide a striking tool to help young professionals face the compelling situations of intraoperative consultation. For cytopathology, to date, cervical screening smears and other materials (e.g., thyroid fine-needle aspirations and effusion cytology) account for a noteworthy percentage of many laboratories’ routine workflows [31]. Nevertheless, the CAP still considered cytopathology as immature for diagnostic DP adoption [24]. Although soon to be released, validation guidelines for employing WSIs for primary diagnosis are still missing [13]. This fact is probably linked to the particular characteristics of cytological specimens and the lack of specifically designed validation studies. As proposed in other recent studies [32], at the time of registration of the current project, the investigators agreed to a consensus-based method to judge the feasibility and safety of WSIs in cytopathology; however, the increasing amount of data that will be progressively collected and shared by the connected hospitals of the network will perfectly suit the need for a broad multicenter study strictly following new guidelines to support the validation of WSIs for cytopathology.

Finally, the huge number of archived WSIs will create large pathology repositories for educational purposes and future technological developments. In particular, a consistent number of linked institutions gathering samples from a highly variable range of inpatient and outpatient services will help to build and expand web-based libraries of pathology fields that are often limited by the scarcity of examples constituting the cohorts (transplantation and neurosurgery, etc.) [33]. This will represent an incredible teaching opportunity for residents, students, and young pathologists to widen their knowledge. Similarly, the easily accessible massive amount of digital data produced will certainly advance the further adoption of AI-guided diagnoses, serving both as external validation datasets for already available algorithms and as the basis for developing new home-built ones.

In conclusion, this proof of concept shows which assessments were made that guided the choices relating to the tools and organizational processes in planning the DP project of the Veneto region. The Veneto DP program aims to transform 12 pathology laboratories from public hospitals producing more than 2.5 million slides per year in a coordinated network of services completely adopting WSI technology. Such an outstanding achievement has been made possible by the integration of all the health information systems of the participating institutions, along with the tireless work of dedicated pathologists, informatics, engineers, and clinicians involved in the project. The preliminary results emerging from the use of the employed LIS, devices, and AI tools firmly confirm and support the safe and effective nature of the entire project.

## Data Availability

All data generated or analyzed during this study are included in this published article.
