# Correction to: Digital pathology structure and deployment in Veneto: a proof-of-concept study

**DOI:** 10.1007/s00428-024-03834-4

**Published:** 2024-05-23

**Authors:** Albino Eccher, Stefano Marletta, Marta Sbaraglia, Angela Guerriero, Mattia Rossi, Giovanni Gambaro, Aldo Scarpa, Angelo Paolo Dei Tos

**Affiliations:** 1 Department of Medical and Sciences for Children and Adults, University of Modena and Reggio Emilia, University Hospital of Modena, Modena, Italy; 2 Department of Diagnostic and Public Health, Section of Pathology, University of Verona, , P.Leee L.A. Scuro N. 10, 37134 Verona, Italy; 3 Division of Pathology, Humanitas Istituto Clinico Catanese, Catania, Italy; 4 Surgical Pathology and Cytopathology Unit, Department of Medicine-DIMED, University of Padua School of Medicine, Padua, Italy; 5 Division of Nephrology, Department of Medicine, University of Verona, Verona, Italy

**Correction to: Virchows Archiv**

10.1007/s00428-024-03823-7

In the published paper, it was observed that the initial letters of the words in the key words section were omitted.

The correct presentation should include: Digital pathology, Automatization, Standardization, Whole slide imaging, Artificial intelligence, Healthcare networks.

The original article has been corrected.

